# Comparative study of commercially available and homemade anti-VAMP7 antibodies using CRISPR/Cas9-depleted HeLa cells and VAMP7 knockout mice

**DOI:** 10.12688/f1000research.15707.2

**Published:** 2019-02-07

**Authors:** Agathe Verraes, Beatrice Cholley, Thierry Galli, Sebastien Nola

**Affiliations:** 1 Membrane Traffic in Health and Disease, INSERM ERL U950, Univ Paris Diderot, Sorbonne Paris Cité, Institut Jacques Monod, CNRS UMR 7592, Paris, France, 75013, France; 2 Membrane Traffic in Healthy & Diseased Brain, Institute of Psychiatry and Neuroscience of Paris, INSERM U1266, Université Paris Descartes, Sorbonne Paris-Cité, 75014, France

**Keywords:** VAMP7, SNARE, monoclonal, polyclonal, CRISPR/Cas9, KO, immunoprecipitation

## Abstract

VAMP7 (vesicle-associated membrane protein) belongs to the intracellular membrane fusion SNARE (Soluble N-ethylmaleimide-sensitive factor attachment protein receptors) protein family. In this study, we used CRISPR/Cas9 genome editing technology to generate VAMP7 knockout (KO) human HeLa cells and mouse KO brain extracts in order to test the specificity and the background of a set of commercially available and homemade anti-VAMP7 antibodies. We propose a simple profiling method to analyze western blotting and use visual scoring for immunocytochemistry staining to determine the extent of the antibodies’ specificity. Thus, we were able to rank the performance of a set of available antibodies and further showed an optimized procedure for VAMP7 immunoprecipitation, which we validated using wild-type and KO mouse brain extracts.

## Introduction

Intracellular membrane fusion in the secretory and endocytic pathways relies on SNARE proteins (soluble N-ethylmaleimide-sensitive factor attachment protein receptors) for membrane fusion events. In order to allow apposition and fusion between two membranes, vesicular (v)- and target (t)-SNARE form a so-called trans-SNARE complex, or SNAREpin. VAMP7 (vesicle associated membrane protein 7) is a clostridial neurotoxin-insensitive v-SNARE that belongs to the “Longin” subfamily (as opposed to the short “Brevins”, like VAMPs 1-3): it encompasses an amino-terminal extension, the Longin domain, which acts as an auto-regulatory domain ^1^. VAMP7 mediates Golgi-derived, late endosomal and lysosomal and autophagosomal related membrane fusion events ^2, 3^ and co-localizes to a large extent with the tetraspanin CD63 ^4, 5^. VAMP7 is involved in exocytosis in several cell types ^6^, including neurons ^7^, in neurotransmitter basal release ^8, 9^ and specific brain circuits and functions ^10^. VAMP7 exocytosis was shown to be regulated by an integrin-, FAK-, and Src-dependent mechanism in developing neurons ^11^ and its transport to the cell periphery by VARP, Rab21 and Kif5 ^7^, while retrograde transport depends on LRRK1 ^12^. In non-neuronal cells, VAMP7 secretory vesicles release compounds such as ATP ^13, 14^ and interleukin-12 ^4^. In addition, VAMP7 regulates trafficking of membrane proteins, including the tetraspanin CD82 ^15^ and the cold-sensing channel TRPM8 ^16^. VAMP7 plays an essential role in cell migration and invasion ^17– 19^. VAMP7 also contributes to the regulation of membrane composition of sphingolipids and GPI-anchored proteins ^20^.

At present date, several antibodies against VAMP7 are commercially available. However, not all of them have been extensively characterized and many reported studies have been conducted using exogenous expression. This is too little information regarding the sensitivity of these antibodies, and may limit their use for super-resolution imaging or proximity ligation assay, which require detection of endogenous proteins.

In this survey, we took advantage of the genome editing CRISPR/Cas9 technology to generate VAMP7-knockout (KO) human HeLa cells. This genetically modified cell line allowed us to test the specificity and background of available commercially or homemade VAMP7 antibodies. Here we compared four mouse monoclonal and four rabbit polyclonal antibodies by western blotting and immunofluorescence using standard protocols. We analyzed the data using a simple profiling of western blots data to extract a specificity index and visually scored immunocytochemistry images in order to rank the performance of the tested antibodies. We further characterized the best ones by immunoprecipitation assays using VAMP7 constructs from different origins and wild type or VAMP7 knockout mouse tissues.

## Material and methods

### Cell culture

HeLa and Cos-7 cells (ATCC CCL-2 and CRL-1651, respectively) were maintained at 37°C and 5% CO _2_ in a humidified incubator, and grown in Dulbecco’s modified Eagle’s medium (DMEM), supplemented with 10% fetal calf serum (FCS), 100 units/ml penicillin, and 100 μg/ml streptomycin (Gibco, Thermo Fisher Scientific). Cells were regularly split using Trypsin-EDTA to maintain exponential growth. Transfection of cells was performed using Lipofectamine 2000 according to the manufacturer's instructions. All culture media reagents were from Thermo Fisher Scientific.

### Generation of VAMP7-depleted cells

Knockout of VAMP7 was achieved using CRISPR RNA-CAS9 guide constructs based on a previously published protocol ^21^. Briefly, sequences for sgRNA were preferentially chosen within the first exon region of VAMP7 genomic gene, with the help of the “RGEN Cas designer” ^22^ web-based tool ( http://rgenome.net/cas-designer/). To limit off-targets, an oligo sequence with ≤2 putative mismatches throughout the whole genome or an ‘out of frame’ score <66 were excluded. The sgRNA target sequences used are: 5’-caccgAACAGCAAAAAGAATCGCCA-3’ (forward) and 5’-aaacTGGCGATTCTTTTTGCTGTTc-3’ (reverse). Oligonucleotides (10 mM) were heated at 95°C for 5 min and annealed by ramping down the temperature from 95°C to 25°C at 5°C min ^-1^. Annealed primers were ligated into pSpCas9(BB)-2A-Puro (PX459) vector (Addgene) using the BbsI sites. After validation by sequencing, the targeting constructs were transfected into HeLa cells following a previously described protocol. An empty pSpCas9(BB)-2A-Puro (PX459) vector was used to generate “control” cells. At 24 h post-transfection, cells were diluted 1/10 and transfected ones were selected by 1 µg/ml puromycin addition for 72 h. The selected populations were then seeded into a 96-well plate at 1 cell per well. Clones derived from single cells were amplified and screened for deficiency by immunoblotting.

### Plasmids

The human and rat GFP-VAMP7 constructs are the same as those that have been described previously ^23^. Plasmid containing mouse VAMP7 cDNA was a kind gift from Maurizio D’Esposito (IGCB, Naples, Italy). Mouse VAMP7 was amplified by PCR and cloned into pEGFP-C3 (Clontech) using HindIII / BamHI restriction sites.

### Mouse colony husbandry and care

The wild-type (WT) and VAMP7 knockout (KO) littermate male cohort was established at the Mouse Clinical Institute animal facility as previously described ^10^. They had a mixed 129/Sv-C57BL/6 genetic background. They were weaned at 4 weeks and housed two to six per M.I.C.E. cage by sex and litter regardless of the genotype under standard conditions and maintained in a room with controlled temperature (21–22°C) under a 12 h light/dark cycle (light on at 7:00 A.M.), with food (standard chow diet, Safe D04) and water available *ad libitum*. All experiments were performed in accordance with the European Communities Council Directive regarding the care and use of animals for experimental procedures (2010/63/UE) and were approved by the local ethical committee (CEEA40-Comité d’Ethique Buffon). Mice were euthanized by cervical dislocation. All efforts were made to ameliorate the suffering of animals and to reduce their number per experiment (1 animal cortex per condition for immunoprecipitation experiment).

### Cortex isolation

The cortex from WT and VAMP7 KO 8 weeks-old C57bl/6 mice were isolated according to a previously published protocol ^10^. Cortex were dissociated by pipette trituration in 500 µl TSE (50 mM Tris-HCl, pH 8.0, 150 mM NaCl, 1 mM EDTA) supplemented with 1% Triton X-100 and cOmplete protease inhibitor tablets (Roche Applied Science) and volume was adjusted in order to get ~2 mg/ml protein final concentration (considering 10 mg of tissue is equivalent to ~1 mg final protein). Lysis was performed by incubation under agitation at 4°C for 30min. After clarification by 16.000 x g centrifugation for 30 min, protein concentration of the supernatant was estimated using Protein Assay Dye Reagent Concentrate (Bio-Rad Laboratories) and immunoprecipitation was carried out (see below).

### Affinity purification

The TG50 (for “Thierry Galli #50”) serum raised against VAMP7 was generated by Covalab (Villeurbanne, France; animal house registration number C21 464 04 EA) using immunization of New Zealand white rabbit with GST-VAMP7 (1-188 aa, as previously described for TG11 ^24^ and TG18 ^25^) and then purified by affinity chromatography. Briefly, serum was clarified and de-lipidated by high-speed centrifugation (70,000 rpm) and applied on a 6xHis-VAMP7 (coiled-coil 1-188 aa) covalently cross-linked HITrap-NHS column (GE Healthcare) overnight at 4°C using a peristaltic pump (0.3 ml/min). We washed the column with filtered and degassed PBS (20 ml, 1 ml/min). Specific antibodies were eluted using 200 mM Glycine/HCl pH 2.2, and collected in tubes containing neutralizing buffer (TBS 10X, 1X final). For each fraction, protein concentration was quantified by optical density (280 nm) measurement.

### Immunoblotting

References for all tested antibodies and reagents used for immunoblotting are listed in Table 1 and Table 2, respectively. Cells were washed in cold phosphate buffered saline (PBS) and lysed 20 min in TSE (50mM Tris-HCl, pH 8.0, 150mM NaCl, 1 mM EDTA) supplemented with 1% Triton X-100 and cOmplete protease inhibitor tablets (Roche Applied Science). Lysates were clarified by centrifugation 30 min at 16.000 x g, and protein concentration was estimated using Protein Assay Dye Reagent Concentrate (Bio-Rad Laboratories). Following heat denaturation at 95°C during 5 min, proteins were separated by 15% SDS-PAGE and transferred to a 0.45-µm nitrocellulose filter (Amersham, GE Healthcare) at 40 mA overnight. Membrane was blocked with 5% low-fat milk in TBS buffer for 20 min at room temperature and probed with indicated primary antibodies diluted in 5% skimmed milk in TBS-T, overnight at 4°C. Following primary antibody incubation, the filter was washed 3× 5 min in TBS-T at room temperature, and probed with HRP- or fluorescently-labeled secondary antibodies diluted in TBS-T for 1 h at room temperature. The filter was then washed 3× 5 min in TBS-T at room temperature. All incubation and washes were performed with gentle rocking. Proteins were detected by enhanced chemiluminescence (ThermoFisher Scientific) and imaged using ImageQuant LAS-4000 (Fujitsu Life Sciences), or scanned in an Odyssey infrared imaging system (Li-Cor).

**Table 1.** Details of primary and secondary antibodies.

Antibody	Origin	Manufacturer	Catalog number	RRID	Stock concentration (mg/ml)	WB dilution	IF dilution
VAMP7 (D4D5J)	Rabbit	Cell Signaling Technology	14811	Not available	0.247	1:800	
VAMP7 (D8Y1R)	Rabbit	Cell Signaling Technology	13876	Not available	0.094		1:200
VAMP7	Rabbit	Sigma-Aldrich	SAB3500844	Not available	1	1:800	1:200
VAMP7	Rabbit	Synaptic Systems	232 003	AB_2212953	1	1:800	1:500
VAMP7 (TG50)	Rabbit	TG lab (affinity purified)	Not relevant	Not relevant	1.5	1:800	1:500
VAMP7 (TG50)	Rabbit	Covalab (Protein A purified)	pab01031-P	Not available	Not tested	Not tested	Not tested
VAMP7	Mouse	Creative Diagnostics	CABT-37960MH	AB_2358849	1	1:800	1:200
VAMP7	Mouse	R&D Systems	MAB6117	AB_10571669	0.5	1:800	1:200
VAMP7 (158.2)	Mouse	TG lab (original clone)	Not relevant	Not relevant	1.5	1:800	1:200
VAMP7 (158.2)	Mouse	Synaptic Systems (recloned 158.2)	232 011	AB_2619947	1.5	1:800	1:200
α-tubulin	Mouse	Sigma-Aldrich	T6074	AB_477582	2	1:800	1:200
GFP	Rabbit	Abcam	ab6673	AB_305643	1		
GFP	Mouse	Roche	11814460001	AB_390913	0.4	1:2000	
Normal IgG	Rabbit	Sigma-Aldrich	I-5006	AB_1163659	1		
Normal IgG	Mouse	Sigma-Aldrich	I-5381	AB_1163670	1		
mouse IgG HRP	Goat	Jackson Immuno Research	115-035-008	AB_2313585	0.8	1:10000	
rabbit IgG HRP	Goat	Jackson Immuno Research	111-035-008	AB_2337937	0.8	1:10000	
mouse IgG IRDye 800	Donkey	Rockland	610-731-124	AB_220145	1	1:5000	
rabbit IgG IRDye 800	Donkey	Rockland	611-731-127	AB_220157	1	1:5000	
mouse AF 488	Goat	Invitrogen	A-11029	AB_2534088	2		1:500
rabbit AF 488	Goat	Invitrogen	A-11034	AB_2576217	2		1:500

**Table 2.** Details of reagents used for immunoblotting.

Process	Reagent	Manufacturer	Catalog number	Concentration/ composition
Sample preparation	Sample reducing agent LDS Sample Buffer	Invitrogen Invitrogen	NP0009 NP0008	10X 4X
Protein ladder	SeeBlue Plus2 Prestained standard	Invitrogen	LC5925	1X
SDS-PAGE Running Buffer	Tris base Glycine SDS	Euromedex Euromedex Euromedex	200923-A 261286405C EU0660	25mM 190mM 0.1% w/v
Protein blotting membrane	0.45µm nitrocellulose transfer membrane			
Wash buffer/ diluent for blocking and antibody (TBS-T)	Tris base NaCl Tween-20	Euromedex VWR Euromedex	200923-A 27810.295 EU0660	20 mM 150 mM 0.1% w/v
Blocking buffer	Milk powder TBS-T			5% w/v 1X
SDS-PAGE Transfer Buffer	Tris base Glycine Ethanol	Euromedex Euromedex VWR	200923-A 261286405C 20824.365	25mM 190mM 20% w/v

### Immunofluorescence staining

The following immunofluorescence staining protocol was performed, with all steps carried out at room temperature unless stated otherwise (see Table 3 for reagent details). HeLa cells were washed once in PBS, fixed with 4% paraformaldehyde in PBS for 20 min, quenched for 20 min with 50 mM NH _4_Cl in PBS and permeabilized by treatment with 0.3% Triton X-100 in PBS for 4 min. After blocking with 10% FCS + 0.3% Triton in PBS for 30 min, cells were incubated overnight with the primary antibodies diluted in 3% FCS + 0.3% Triton in PBS at 4°C. After several washes with 3% FCS, 0.3% Triton in PBS, cells were then incubated with the secondary antibodies in 3% FCS, 0.3% Triton in PBS for 1 h, then washed several times in 0.3% Triton in PBS. Coverslips were partially dried and mounted in Prolong medium (Invitrogen), and then left to set overnight. Fluorescence microscopy and imaging were performed using an upright epifluorescence microscope (DMRA2, Leica Microsystem) equipped with a CMOS camera (Orca Flash 4.0 LT, Hamamatsu) and a HCX PL APO 100x/1.40-0.70 oil CS oil-immersion Leica objective. Dilution of primary antibodies ( Table 1) was formerly optimized to get relatively equivalent signal intensity in the WT cells for the same microscope settings (time of exposure, binning, objective, etc.), allowing direct comparison of signal between antibodies.

**Table 3.** Details of reagents used for immunofluorescence.

Process	Reagent	Manufacturer	Catalog number	Concentration/ Composition
Washing	PBS	Home-made		1x
Fixing	Paraformaldehyde	Prolabo	PRO-28794.295	4% in PBS 1x
Permeabilizing, Washing (PBS-T)	PBS Triton X100	Home-made Merck	9410	1x 0.3% w/v
Blocking	Fetal Bovine Serum (FBS) PBS-T	Thermofisher	10270106	10% v/v
Antibody diluent	Fetal Bovine Serum (FBS) PBS-T	Thermofisher	10270106	3% v/v
Nuclear staining	DAPI (4’,6-diamidino-2- phenylindole)	Invitrogen	D3571	0.2µg/ml in PBS
Mounting	Prolong antifade	Invitrogen	P36930	1X

### Immunoprecipitation

Transfected Cos7 cells were washed once in PBS 1X then lysed as described in the “immunoblotting” section. Immunoprecipitation experiments were carried out as followed (see Table 4 for reagent details). Briefly, 1 mg of protein extract was submitted to immunoprecipitation by incubation overnight at 4°C with 2.5 µg of antibodies that were pre-coupled with 25 μl magnetic beads (Dynabeads M-280, Invitrogen). Beads were then extensively washed with TSE-1% Triton and beads resuspended in 2X Laemmli buffer. Samples were loaded on 4–12% Bis-Tris NuPAGE (ThermoFisher Scientific) or RunBlue SDS (Expedeon) gels with manufacturer-recommended electrophoresis buffer, processed for western blotting using HRP-coupled secondary antibodies and enhanced chemiluminescence (ThermoFisher Scientific).

**Table 4.** Details of reagents used for immunoprecipitation.

Process	Reagent	Manufacturer	Catalog number	Concentration/ Composition
Magnetic Coupling	Dynabeads M-280 Sheep anti-mouse IgG Dynabeads M-280 Sheep anti-rabbit IgG	Invitrogen Invitrogen	11201D 11203D	10mg/ml 10mg/ml
Washing (TSE-T)	Tris HCl pH8.0 NaCl EDTA Triton X100	Sigma VWR Eurobio Merck	T2694 27810.295 GAUEDT0064 9410	50 mM 150 mM 1 mM 1% w/v
Sample preparation	Sample reducing agent LDS Sample Buffer	Invitrogen Invitrogen	NP0009 NP0008	10X 4X

For immunoprecipitation of endogenous VAMP7, 1 mg of mouse cortex extracts (see “Cortex isolation” section) were submitted to immunoprecipitation as for cell extracts, excepted that 5 µg of antibodies, 40 μl magnetic beads (Dynabeads M-280, Invitrogen), fluorescent secondary antibodies and an Odyssey infrared imaging system (LI-COR, Lincoln, Nebraska, USA) were used.

### Quantitative analysis and scoring

All quantification analyses was performed using ImageJ (1.49n) and data were computed in Microsoft Excel.

**Figure 1.**
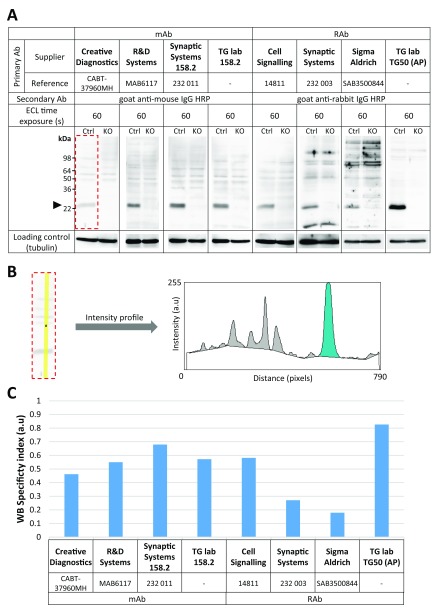
Comparative immunoblot analysis of endogenous VAMP7 level of expression in control or CRISPR/Cas9-modified VAMP7-depleted HeLa cells. ( **A**) Immunoblot performed on lysates from control (Ctrl) and VAMP7 knockout (KO) HeLa cells. An equal amount of total protein extracts from each condition was run in replicates. Following transfer on nitrocellulose and blocking, membrane was sliced and each piece was probed with indicated anti-VAMP7 antibodies (expected size: ~25 kDa). Time of exposure for each condition is provided. For loading control, membrane was washed and incubated with anti-α-tubulin antibody (expected size: ~50 kDa). ( **B**) Example of quantification of western blotting signal from ( **A**) (see dotted red line). Intensity profile (right panel) was generated from a 20-pixel-wide straight line (yellow, left panel) across each lane. On the intensity profile (right panel), areas corresponding to the VAMP7 signal and the non-specific bands are shown in blue and gray, respectively (see Methods section for details). ( **C**) Quantification of each antibody tested by western blotting shown in ( **A**). The “specificity index” represents the signal-to-noise ratio of the antibodies, reflecting the intensity of the VAMP7 band amongst the overall signal per lane (including non-specific bands) and its specificity in the control condition compared to KO (see Methods section for details). AP, affinity purified.

For western blotting signal quantification, a 20-pixel-wide straight line vertically crossing each lane was drawn to generate an intensity profile (see Figure 1A and B, left panel). Local background correction was performed by manually drawing a segmented line under the peaks representing the bands detected by western blotting (Figure B, right panel). The VAMP7 band was defined as the ~25 kDa band that would disappear or diminish in intensity in the KO extract compared with the WT extract. Areas under all peaks and the VAMP7 one, shown in grey and blue, respectively ( Figure 1B, right panel), were measured. In order to estimate the signal-to-noise ratio of each antibody, taking into account the intensity of the band of interest over the intra-lane non-specific ones and the specificity of the signal in the control condition versus the KO ones, a so-called “WB specificity index” was calculated for each lane of the western blot, using the following equation:

$$\text{WB}\;\text{specificity}\;\text{index}={\left\{\frac{{\text{Area}}_{\text{(peak}\;\text{of}\;\text{interest)}}}{{\text{Area}}_{\text{(all}\;\text{peaks)}}}\right\}}_{\text{control}}-{\left\{\frac{{\text{Area}}_{\text{(peak}\;\text{of}\;\text{interest)}}}{{\text{Area}}_{\text{(all}\;\text{peaks)}}}\right\}}_{\text{VAMP7}\;\;\text{KO}}$$

For immunocytochemistry, we visually scored the specificity of each antibodies based on two features : i) “typical VAMP7 localization pattern” which is defined as intracellular punctate membrane pattern with perinuclear concentration and peripheral vesicles, ii) “loss of signal in KO” which corresponds to clear reduction of signal in the KO compared to WT condition, similar to the intensity of the “secondary-only” condition. Thus, the best antibodies to be used for endogenous staining of VAMP7 in Hela cells were positively scored (“+”) for each criteria when it was obviously met.

## Results

### Tools development and description of antibodies tested in this study

In order to characterize and compare a set of commercially available and homemade (i.e from “Thierry Galli’s lab”, hereafter referred as “TG lab”) anti-VAMP7 antibodies ( Table 1), we first generated VAMP7 knockout cells, using CRISPR/Cas9 engineering ^21^, as described in the methods section. HeLa cells were chosen as they express VAMP7 endogenously in a detectable amount by western blotting and immunocytochemistry ^15^.

Some of the tested antibodies were described in the provider’s datasheet to only work for immunofluorescence detection (e.g. Cell Signaling, catalogue number 13876, clone D81Y1R) or western blotting (e.g. Cell Signaling, catalogue number 14811, clone D4D5J) and were used accordingly. Our lab generated two antibodies, the mouse monoclonal “158.2” ^26^ and the rabbit polyclonal “TG50”, which are commercially available from Synaptic Systems and Covalab (TG50 as protein A purified serum), respectively. Only the in-house affinity-purified (AP) version of the TG50 antibody was included in this study because we wanted an affinity-purified serum as best possible positive control.

### Characterization of antibodies by western blotting

We compared four monoclonal and four polyclonal rabbit antibodies by western blotting using control or VAMP7-KO HeLa cell extracts ( Figure 1). We used an anti-tubulin antibody as loading control. All antibodies tested in the described conditions ( Table 2) were sensitive enough to detect a prominent band at the expected molecular weight (~25 kDa) in the control condition. This band was absent in the VAMP7-KO cell lines in all cases. However, some non-specific bands were visible for all the tested antibodies in both control and VAMP7-KO cell lysates, particularly with polyclonal Synaptic Systems (catalogue number 232 003) and Sigma-Aldrich (catalogue number T6074) antibodies. Therefore, all the tested antibodies showed a signal that was specific for VAMP7, but they also showed variable background bands. As assessed by intensity profile analysis ( Figure 1B) and our western blotting specificity index ( Figure 1C), the TG lab (TG50) antibody showed the best signal-to-noise ratio using this Western blotting conditions, which may not be surprising, because it had been affinity-purified.

### Characterization of antibodies by immunofluorescence

**Figure 2.**
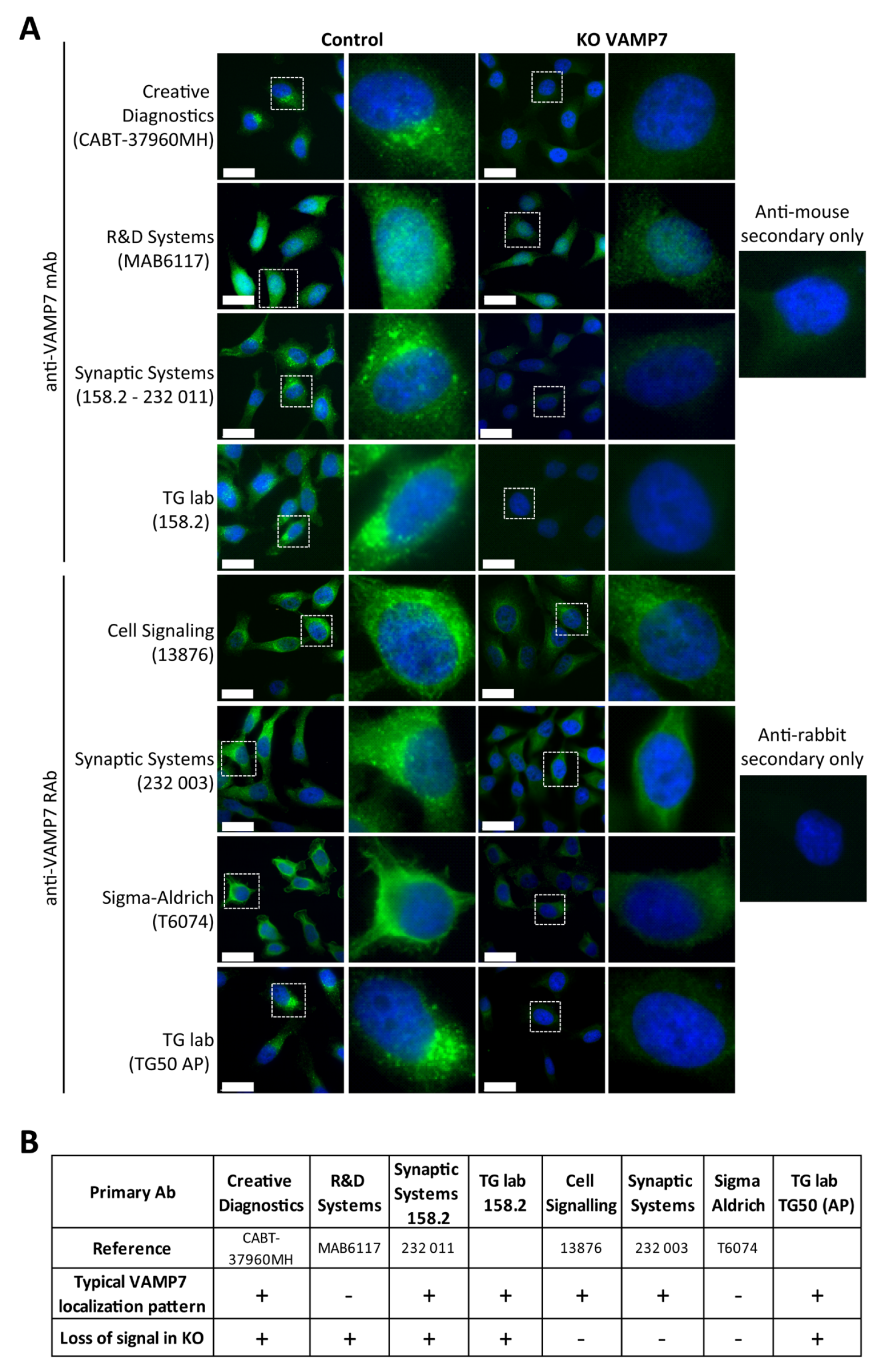
Comparative immunostaining of VAMP7 endogenous level of expression in control or depleted HeLa cells. ( **A**) Control and VAMP7 knockout (KO) HeLa cells were grown on glass coverslips, fixed in paraformaldehyde, blocked and immunostained with the indicated mouse or rabbit anti-VAMP7 antibodies (green) and DAPI (blue). KO condition allows the estimation of background signal. Samples were imaged with an epifluorescence microscope using a 100X objective. Bars, 25 µm. ( **B**) Table recapitulating visual scoring of indicated antibodies used for immunostaining. Antibodies were scored for yielding a “typical VAMP7 localization pattern” (ie intracellular punctate membranes pattern with perinuclear concentration and peripheral vesicles) and “loss of signal in KO”. Characters used in the table indicated whether the above-mentioned criteria are obviously met (“+”) or not (“-”). AP, affinity purified.

In order to better characterize these antibodies ( Table 1), we performed immunostaining in control and VAMP7 KO HeLa cells. For this assay, the rabbit antibody from Cell Signalling Technology clone D8Y1R (ref. 13876) was used instead of the D4D5J clone (ref. 14811), according to the manufacturer’s recommendations. To compare the specificity of the antibodies, we adjusted their dilution ( Table 1) to get relatively equivalent signal intensity in the WT cells with the same acquisition time on the microscope. According to this assay, the mouse antibodies from Creative Diagnostics (CABT-37960MH), Synaptic Systems (158.2–232 011), TG lab (158.2) and the rabbit antibodies Synaptic Systems (232 003) and TG lab (TG50) stained perinuclear membrane structures and vesicles dispersed in the cytoplasm, a typical and already described localization pattern for VAMP7 in HeLa cells ^10, 15, 27^. However, in the WT HeLa cells, the R&D Systems (MAB6117) antibody gave a homogenous signal, which spread into the nucleus, the Cell Signaling (14811) antibody, seemed to also stain perinuclear ER-like structures and the Sigma-Aldrich (T6074) antibody exhibited a diffuse cytoplasmic pattern with an absence of vesicular staining. The majority of the tested antibodies seemed to show an overall lower-intensity signal in the VAMP7 KO cells compared to control. According to the “secondary-only” condition that reveals the internal background signal of the experiment ( Figure 2A, right panel) and the scoring analysis we conducted ( Figure 2B, see Methods for details), the most reliable antibodies to be used for endogenous staining of VAMP7 in Hela cells are the Creative Diagnostics (CABT-37960MH), Synaptic Systems (232 003) and TG lab (158.2 and TG50) antibodies.

### Characterization of antibodies by immunoprecipitation

**Figure 3.**
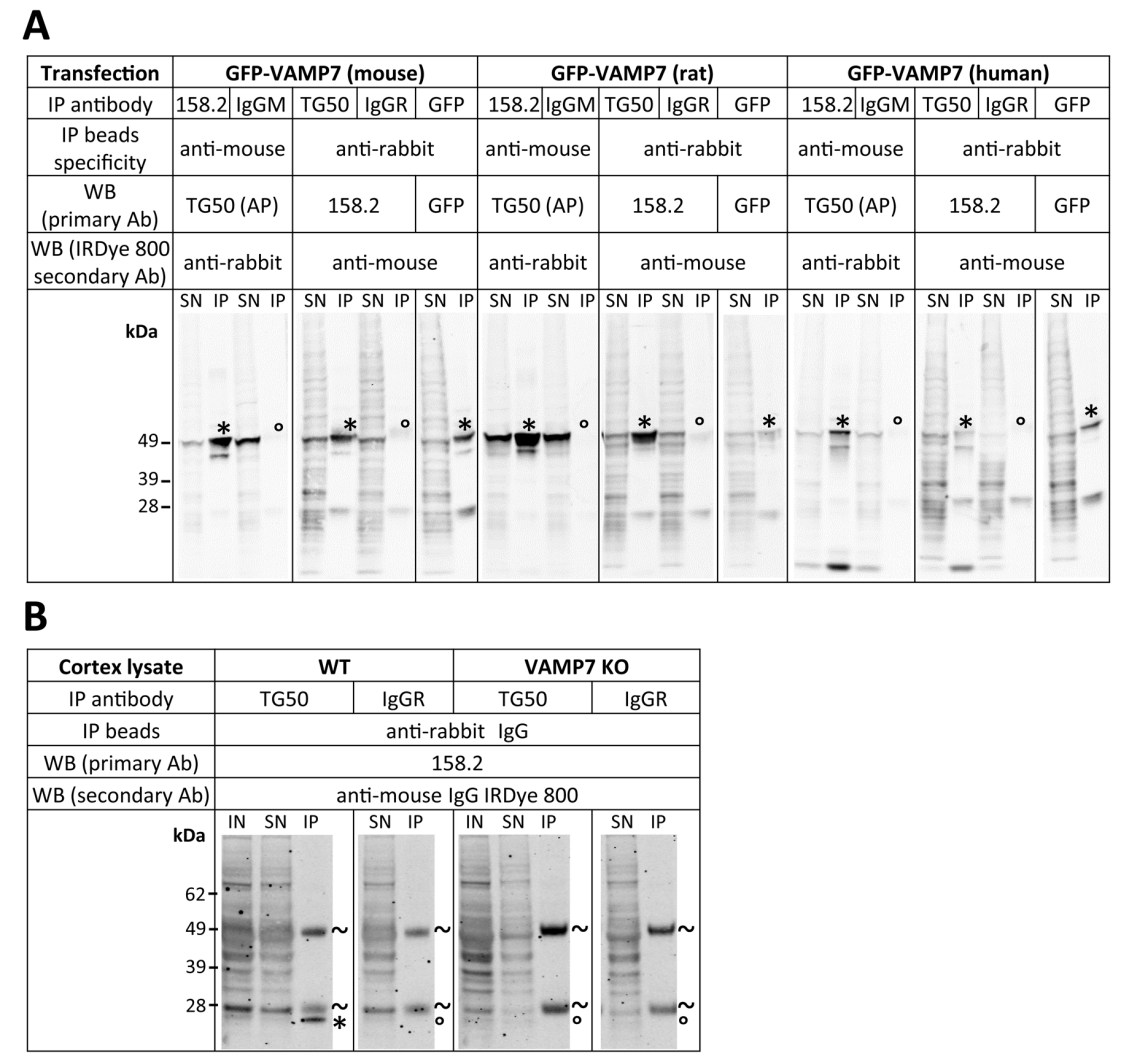
Comparison of available anti-VAMP7 antibodies for exogenous and endogenous immunoprecipitation. ( **A**) Cos cells overexpressing GFP-tagged mouse, rat or human VAMP7 constructs were lysed and VAMP7 was immunoprecipitated using indicated specific antibodies (“IP Antibody”). Normal isotype IgG (“IgGM” or “IgGR”) and GFP antibody were used as negative and positive control, respectively. Supernatants after immunoprecipitation (SN) and immunoprecipitates (IP) were probed with indicated VAMP7 antibodies (WB primary Ab). ( **B**) VAMP7 was immunoprecipitated from wild-type (“WT”) and VAMP7 knock-out (KO) mouse cortex lysates using rabbit anti-VAMP7 TG50 antibody and detected with mouse anti-VAMP7 158.2. Normal isotype IgG (IgGR) were used as negative control. Inputs (IN), supernatants after immunoprecipitation (SN) and immunoprecipitates (IP) were probed with mouse anti-VAMP7 158.2 antibody. *VAMP7 band of interest (expected size of GFP-VAMP7 constructs: ~50 kDa). °Absence of band at expected size. ~heavy and light chains of the antibody used for immunoprecipitation. AP = affinity purified.

Taken together, immunoblot and immunofluorescence assays suggest that the homemade anti-VAMP7 antibodies (158.2 and TG50) showed the best endogenous signal-to-noise ratio in HeLa cells. The immunoprecipitation assays were limited to these two homemade antibodies as some commercial antibodies are not supplied at a high enough concentration to be conveniently used in such experiments. To check the inter-species specificity of these antibodies, we carried out immunoprecipitation assays in Cos cells overexpressing mouse, rat or human GFP-tagged VAMP7 constructs ( Figure 3A). We used 158.2 and TG50 for immunoprecipitation and immunoblot and species-specific IgG and anti-GFP antibodies as negative and positive controls for immunoprecipitation, respectively ( Table 4). Both 158.2 and TG50 antibodies exhibited a sharp ~50 kDa band in all immunoprecipitation lanes, demonstrating a relatively equivalent ability to precipitate either mouse, rat or human VAMP7, while a very faint signal was observed in the negative control IgG IP lanes.

We next wondered whether or not these antibodies could immunoprecipitate endogenous VAMP7 from tissue and test background signal in KO tissue. To this aim, we chose to perform immunoprecipitation on lysates from WT or VAMP7 KO mouse cortex using the rabbit TG50 antibody because it performed the best in previous assays and to observe immunoprecipitated VAMP7 with the mouse 158.2 antibody ( Figure 3B). Although 158.2 antibody showed stronger background and multiple bands compared to the pattern seen in HeLa cells ( Figure 1), a clear band at the expected molecular weight (~25 kDa) was present for the WT immunoprecipitation condition but not VAMP7 KO, demonstrating the specificity and low background noise of the TG50 antibody for immunoprecipitation of endogenous VAMP7 in mouse tissue extracts. Altogether, we showed here that both 158.2 and TG50 antibodies were able to immunoprecipitate VAMP7 from different species and that immunoprecipitation of endogenous VAMP7 could be performed in mouse tissue extracts using TG50 for IP and 158.2 (or TG50, see Dataset 3) for subsequent western blotting.

Raw images of experimental replicates for Figure 1, immunoblotting experiments This dataset includes uncropped blots for all experimental replicates that are represented in Figure 1. Treatments and immunoblot methods were performed as outlined in Figure 1. Blots were probed with indicated anti-VAMP7 antibodies and anti-α-tubulin antibodies was used as a loading control. (A) Dataset used for Figure 1, with cropped regions in red dashed line. (B) Additional set of raw images of a replicate experiment. Quantification as performed in Figure 1 is shown in lower panel. Note that although signal intensity and background are different within these two replicates, the relative performance of the different tested antibodies remained the same. Click here for additional data file. Copyright: © 2019 Verraes A et al. 2019 Data associated with the article are available under the terms of the Creative Commons Zero "No rights reserved" data waiver (CC0 1.0 Public domain dedication).

Raw images of additional experimental replicates for Figure 2, immunofluorescence experiments This dataset includes additional images from experimental replicates of the images presented in Figure 2. Immunofluorescence staining was performed as described for Figure 2. Images were taken at 40× objective. Bar, 15µm. Click here for additional data file. Copyright: © 2019 Verraes A et al. 2019 Data associated with the article are available under the terms of the Creative Commons Zero "No rights reserved" data waiver (CC0 1.0 Public domain dedication).

Raw images of immunoprecipitation experiments for Figure 3, immunoprecipitation Uncropped data from Figure 3 (A) and replicate (C) for VAMP7 immunoprecipitation from Cos-7 cell lysate overexpressing GFP-tagged mouse, rat or human VAMP7 constructs. Uncropped immunoblotting data from Figure 3 (B) and replicate (D) for VAMP7 immunoprecipitation from WT and VAMP7 KO mouse cortex extracts. Antibodies used for immunoprecipitation and subsequent immunoblotting are indicated. Red dashed lines show GFP-VAMP7 protein and cropped region, respectively. IN=Input (50 µg in A and C, 100 µg in B and D); SN = supernatant after immunoprecipitation; IP = immunoprecipitate; * = GFP-VAMP7; ° = Absence of band at GFP-VAMP7 size (~50 kDa); ~: immunoglobulins. Click here for additional data file. Copyright: © 2019 Verraes A et al. 2019 Data associated with the article are available under the terms of the Creative Commons Zero "No rights reserved" data waiver (CC0 1.0 Public domain dedication).

## Discussion

In this antibody survey, we used genome-edited VAMP7 KO HeLa cells to compare several commercially available and homemade antibodies using standard methods for western blotting and immunocytochemistry. Using optimal staining protocol for each commercially available antibody might have been an excellent option to compare antibodies, although i) not all antibodies datasheet clearly detail the best conditions to use which would have led to very time-consuming round of optimizations, ii) except for the HeLa cells that would have been similar between conditions, every other parameters would have been different, a situation which we found more likely to lead to questionable conclusions regarding our comparative study of antibodies. In addition, as mentioned in Table 1, some antibodies are delivered at low concentrations thus an optimization testing of a wide range of conditions would be very costly for any end-user. Apart from poor reactivity/quality, high background observed in western blotting or non-specific signal of some tested antibodies in IF could be due to non-fully optimized technical procedures. For example, different blocking agent could be used, such as bovine serum albumin for immunoblot or immunofluorescence assays, or cells could have been fixed differently (methanol, glutaraldehyde). We also cannot totally exclude some batch effect for the poor signal observed with some commercial antibodies. Furthermore, we voluntarily restricted this survey to the VAMP7-depleted HeLa cell line generated in this study and tissues from KO mice. We chose to evaluate the background signal of these antibodies in KO cells, criteria of choice that is both crucial for any assay and not always convincingly characterized. Conducting the same study in a different cell type or species might have led to different conclusions regarding background and specificity. Although it might have reduced the overall background, particularly in the VAMP7 KO immunofluorescence conditions, we chose to use epifluorescence microscopy rather than confocal imaging in order to give a global overview of the signal obtained with these antibodies and because many studies still largely rely on wide field microscopy. With these words of caution and limitations, we conclude that 158.2 and TG50 antibodies appeared as the best performers in our assays.

The datasheet provided with the Synaptic Systems (158.2–232 011) antibody indicates that it is specific for rat and mouse. Here we provide evidence that it is also able to specifically recognize VAMP7 in human HeLa cells, both in immunoblot and immunofluorescence assays. This is in good agreement with the fact that human, rat and mouse VAMP7 protein sequences share more than 94% identity (alignment with www.uniprot.org website). MAb 158.2 also immunoprecipitated mouse, rat and human GFP-tagged VAMP7 ( Figure 3A). Altogether, 158.2 and TG50 thus appeared as suitable antibodies in all species and for all applications. However, 158.2 antibody may not be very sensitive, as it did not allow for the endogenous detection of low amounts of the protein, particularly in tissues ( Figure 3) while TG50 performed slightly better ( Dataset 3). More generally, further comparative study should be conducted to formally assess the efficiency of this set of antibodies in non-human cell lines or tissues, particularly in immunocytochemistry. However, immunoprecipitation using TG50 (protein A purified serum available from Covalab, ref. pab01031-P) and 158.2 or TG50 ( Dataset 3) detection appeared as a valid strategy for specific isolation of the endogenous VAMP7.

Finally, we proposed here an easy profile comparison of WT and KO western blotting signals and visual scoring criteria for immunocytochemistry staining in order to rank the quality of antibodies directed against membrane-associated proteins as a decision-making tool for more complex studies.

## Data availability

The data referenced by this article are under copyright with the following copyright statement: Copyright: © 2019 Verraes A et al.

Data associated with the article are available under the terms of the Creative Commons Zero "No rights reserved" data waiver (CC0 1.0 Public domain dedication). 

**Dataset 1. Raw images of experimental replicates for Figure 1, immunoblotting experiments.** This dataset includes uncropped blots for all experimental replicates that are represented in Figure 1. Treatments and immunoblot methods were performed as outlined in Figure 1. Blots were probed with indicated anti-VAMP7 antibodies and anti-α-tubulin antibodies was used as a loading control. (A) Dataset used for Figure 1, with cropped regions in red dashed line. (B) Additional set of raw images of a replicate experiment. Quantification as performed in Figure 1 is shown in lower panel. Note that although signal intensity and background are different within these two replicates, the relative performance of the different tested antibodies remained the same. DOI: http://dx.doi.org/10.5256/f1000research.15707.d221360 ^28^.

**Dataset 2. Raw images of additional experimental replicates for Figure 2, immunofluorescence experiments.** This dataset includes additional images from experimental replicates of the images presented in Figure 2. Immunofluorescence staining was performed as described for Figure 2. Images were taken at 40× objective. Bar, 15µm. DOI: http://dx.doi.org/10.5256/f1000research.15707.d234810 ^29^.

**Dataset 3. Raw images of immunoprecipitation experiments for Figure 3, immunoprecipitation.** Uncropped data from Figure 3 (A) and replicate (C) for VAMP7 immunoprecipitation from Cos-7 cell lysate overexpressing GFP-tagged mouse, rat or human VAMP7 constructs. Uncropped immunoblotting data from Figure 3 (B) and replicate (D) for VAMP7 immunoprecipitation from WT and VAMP7 KO mouse cortex extracts. Antibodies used for immunoprecipitation and subsequent immunoblotting are indicated. Red dashed lines show GFP-VAMP7 protein and cropped region, respectively. IN=Input (50 µg in A and C, 100 µg in B and D); SN = supernatant after immunoprecipitation; IP = immunoprecipitate; * = GFP-VAMP7; ° = Absence of band at GFP-VAMP7 size (~50 kDa); ~: immunoglobulins. DOI: http://dx.doi.org/10.5256/f1000research.15707.d234809 ^30^.
