# Supplementary material for: Comparative study of commercially available and homemade anti-VAMP7 antibodies using CRISPR/Cas9-depleted HeLa cells and VAMP7 knockout mice
Source: F1000Res. 2019 Feb 7;7:1649. Originally published 2018 Oct 16. [Version 2] doi: 10.12688/f1000research.15707.2 (PMC6376254; doi:10.12688/f1000research.15707.2)
Supplement: Raw images of experimental replicates for Figure 1, immunoblotting experiments — This dataset includes uncropped blots for all experimental replicates that are represented in Figure 1. Treatments and immunoblot methods were performed as outlined in Figure 1. Blots were probed with indicated anti-VAMP7 antibodies and anti-α-tubulin antibodies was used as a loading control. (A) Dataset used for Figure 1, with cropped regions in red dashed line. (B) Additional set of raw images of a replicate experiment. Quantification as performed in Figure 1 is shown in lower panel. Note that although signal intensity and background are different within these two replicates, the relative performance of the different tested antibodies remained the same. [file f1000research-7-19822-s0000.tgz › e4981809-12d1-4796-9c71-7047d3d7ab18_dataset1.pdf]

Dataset 1. Raw images of immunoblotting experiments for Figure 1

A

Primary Ab	Supplier	**Creative Diagnostics**	**Synaptic Systems 158.2**	**TG lab 158.2**		**Cell Signalling**		**R&D Systems**
	Reference	CABT-37960MH	232 011	-		14811		MAB6117
		WT | KO	WT | KO	WT | KO		WT | KO		WT | KO

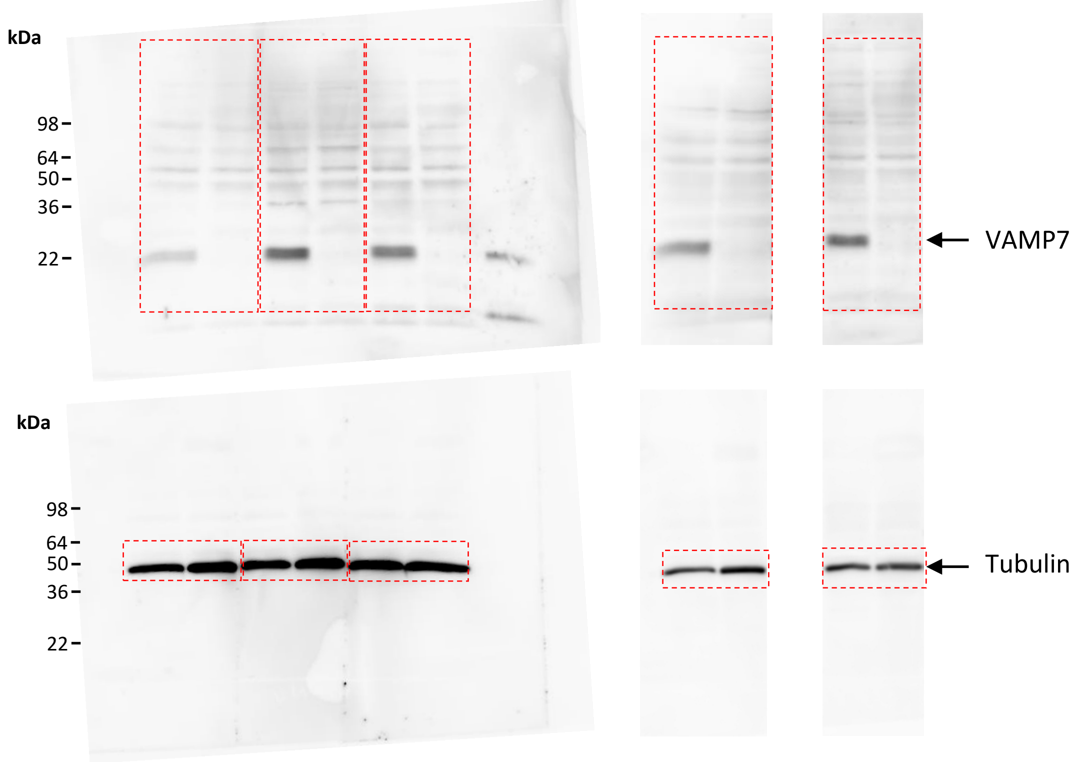

Primary Ab	Supplier	**Synaptic Systems**		**Sigma Aldrich**		**TG Lab TG50 (AP)**
	Reference	232 003		SAB3500844		
		WT | KO		WT | KO		WT | KO

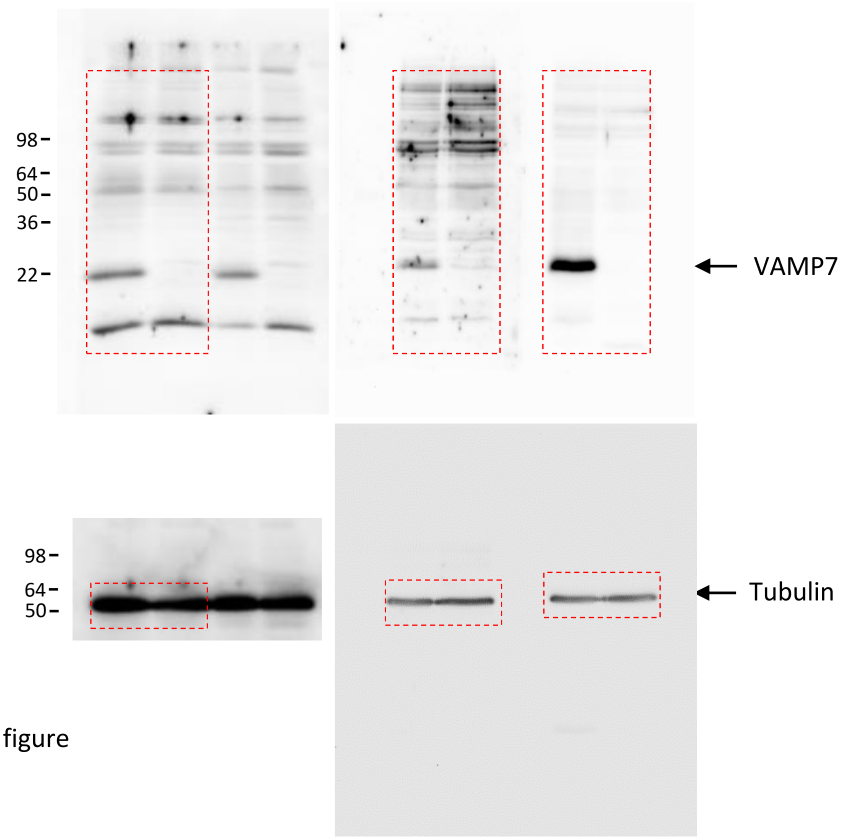

: area displayed on the figure

Dataset 1. Additional raw images of immunoblotting experiments for Figure 1

B

		mAb					RAb				
Primary Ab	Supplier	Creative Diagnostics	R&D Systems	Synaptic Systems 158.2	TG lab 158.2		Cell Signalling	Synaptic Systems	Sigma Aldrich		TG lab TG50 (AP)
	Reference	CABT-37960MH	MAB6117	232 011	-		14811	232 003	SAB3500844		-
		WT KO	WT KO	WT KO	WT KO		WT KO	WT KO	WT KO		WT KO

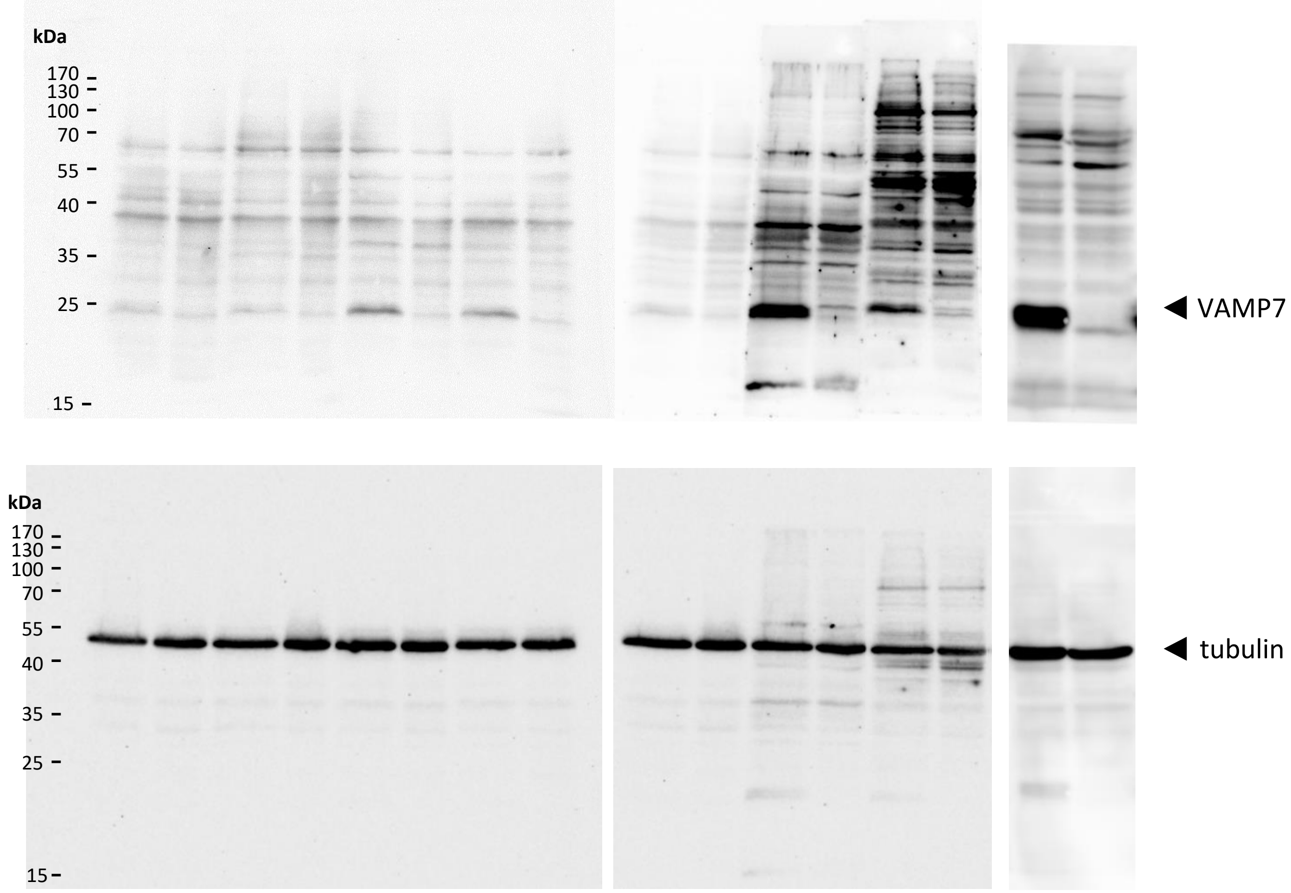

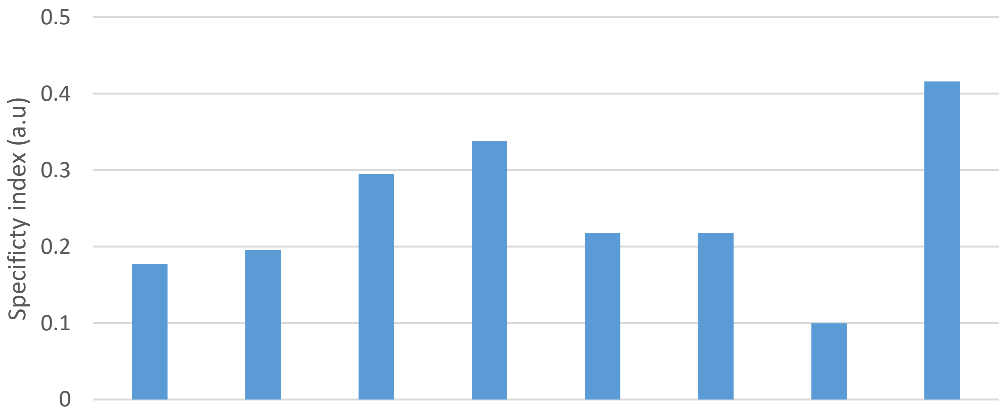

Creative Diagnostics	R&D Systems	Synaptic Systems 158.2	TG lab 158.2	Cell Signalling	Synaptic Systems	Sigma Aldrich	TG lab TG50 (AP)
CABT-37960MH	MAB6117	232 011	-	14811	232 003	SAB3500844	-
