# Supplementary material for: Comparative study of commercially available and homemade anti-VAMP7 antibodies using CRISPR/Cas9-depleted HeLa cells and VAMP7 knockout mice
Source: F1000Res. 2019 Feb 7;7:1649. Originally published 2018 Oct 16. [Version 2] doi: 10.12688/f1000research.15707.2 (PMC6376254; doi:10.12688/f1000research.15707.2)

Dataset 2. Raw images of additional experimental replicates for Figure 2, immunofluorescence experiments.

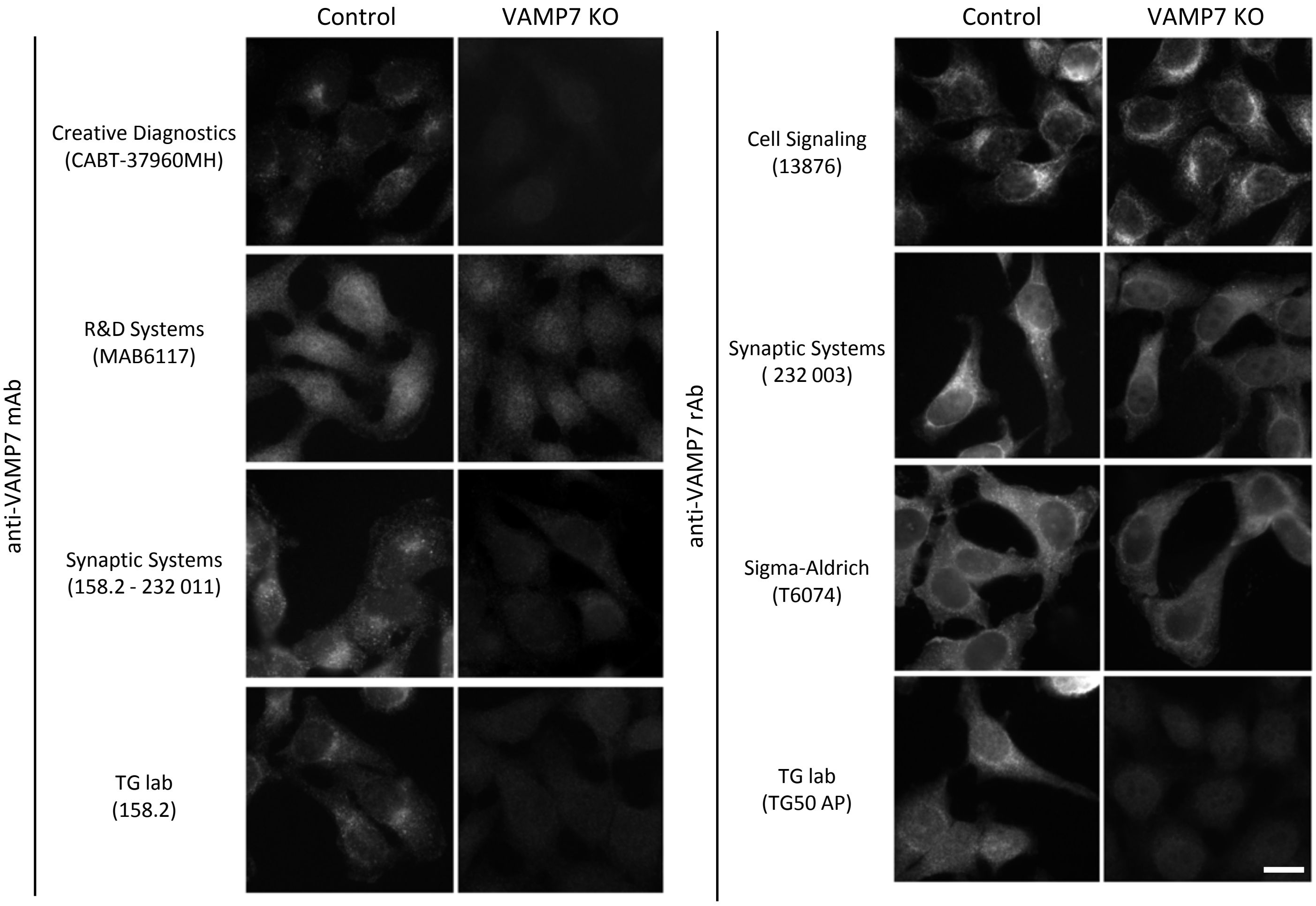

Supplement: Raw images of additional experimental replicates for Figure 2, immunofluorescence experiments — This dataset includes additional images from experimental replicates of the images presented in Figure 2. Immunofluorescence staining was performed as described for Figure 2. Images were taken at 40× objective. Bar, 15µm. [file f1000research-7-19822-s0001.tgz › 595684be-2cfa-472b-aae3-6f6ff1d82cb4_Dataset_2_v2.pdf]
