# Supplementary material for: Comparative study of commercially available and homemade anti-VAMP7 antibodies using CRISPR/Cas9-depleted HeLa cells and VAMP7 knockout mice
Source: F1000Res. 2019 Feb 7;7:1649. Originally published 2018 Oct 16. [Version 2] doi: 10.12688/f1000research.15707.2 (PMC6376254; doi:10.12688/f1000research.15707.2)
Supplement: Raw images of immunoprecipitation experiments for Figure 3, immunoprecipitation — Uncropped data from Figure 3 (A) and replicate (C) for VAMP7 immunoprecipitation from Cos-7 cell lysate overexpressing GFP-tagged mouse, rat or human VAMP7 constructs. Uncropped immunoblotting data from Figure 3 (B) and replicate (D) for VAMP7 immunoprecipitation from WT and VAMP7 KO mouse cortex extracts. Antibodies used for immunoprecipitation and subsequent immunoblotting are indicated. Red dashed lines show GFP-VAMP7 protein and cropped region, respectively. IN=Input (50 µg in A and C, 100 µg in B and D); SN = supernatant after immunoprecipitation; IP = immunoprecipitate; * = GFP-VAMP7; ° = Absence of band at GFP-VAMP7 size (~50 kDa); ~: immunoglobulins. [file f1000research-7-19822-s0002.tgz › 339e56e0-8e02-4008-a572-5b3373396214_Dataset3_v2.pdf]

Dataset 3. Raw images of immunoprecipitation experiments for Figure 3

A

Transfection	GFP-VAMP7 (mouse)				
IP antibody	158.2	IgGM	TG50	IgGR	GFP
IP beads specificity	anti-mouse		anti-rabbit		
WB (primary Ab)	TG50 (AP)		158.2		GFP
WB (IgG IRDye 800 secondary Ab)	anti-rabbit		anti-mouse		

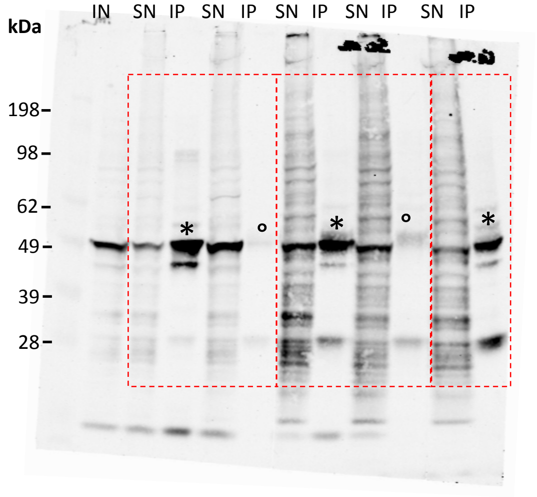

Transfection	GFP-VAMP7 (rat)				
IP antibody	158.2	IgGM	TG50	IgGR	GFP
IP beads specificity	anti-mouse		anti-rabbit		
WB (primary Ab)	TG50 (AP)		158.2		GFP
WB (IgG IRDye 800 secondary Ab)	anti-rabbit		anti-mouse		

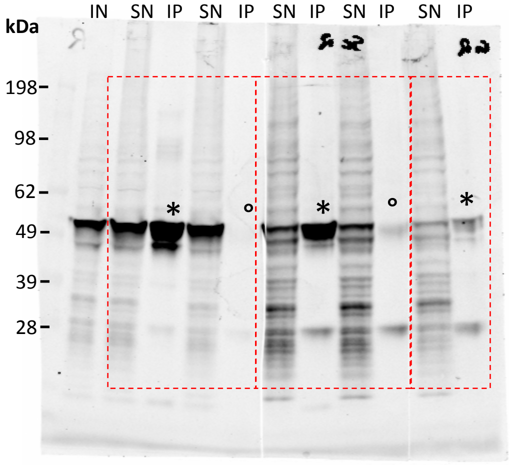

* = GFP-VAMP7 (expected size: ~50 kDa)

° = Absence of band at GFP-VAMP7 size

Transfection	GFP-VAMP7 (human)				
IP antibody	158.2	IgGM	TG50	IgGR	GFP
IP beads specificity	anti-mouse		anti-rabbit		
WB (primary Ab)	TG50 (AP)		158.2		GFP
WB (IgG IRDye 800 secondary Ab)	anti-rabbit		anti-mouse		

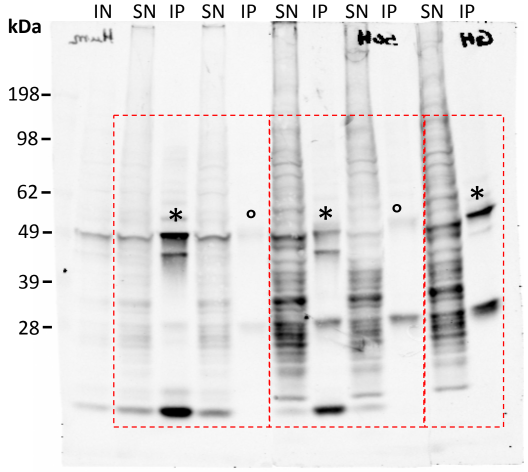

: area displayed on the figure

B

Cortex lysate	WT						VAMP7 KO				
IP antibody		TG50		IgGR				TG50		IgGR	
IP beads	anti-rabbit IgG										
WB (primary Ab)	158.2										
WB (secondary Ab)	anti-mouse IgG IRDye 800										
	IN	SN	IP	SN	IP		IN	SN	IP	SN	IP

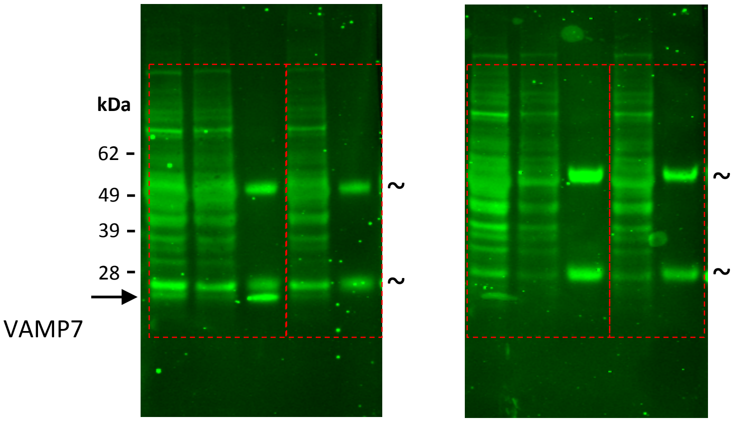

~: immunoglobulins

Dataset 3. Additional raw images of immunoprecipitation experiments for Figure 3

C

Transfection	GFP-VAMP7 (mouse)				
IP antibody	158.2	IgGM	TG50	IgGR	GFP
IP beads specificity	anti-mouse		anti-rabbit		
WB (primary Ab)	TG50 (AP)		158.2		GFP
WB (IgG IRDye 800 secondary Ab)	anti-rabbit		anti-mouse		

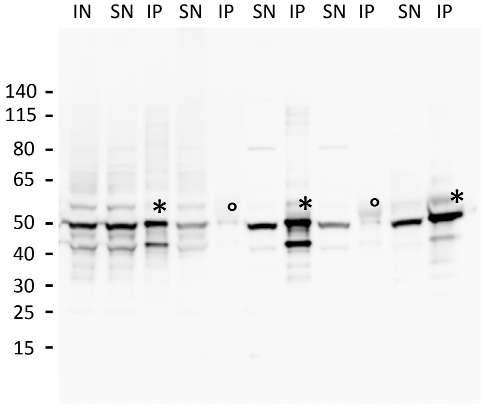

Transfection	GFP-VAMP7 (rat)				
IP antibody	158.2	IgGM	TG50	IgGR	GFP
IP beads specificity	anti-mouse		anti-rabbit		
WB (primary Ab)	TG50 (AP)		158.2		GFP
WB (IgG IRDye 800 secondary Ab)	anti-rabbit		anti-mouse		

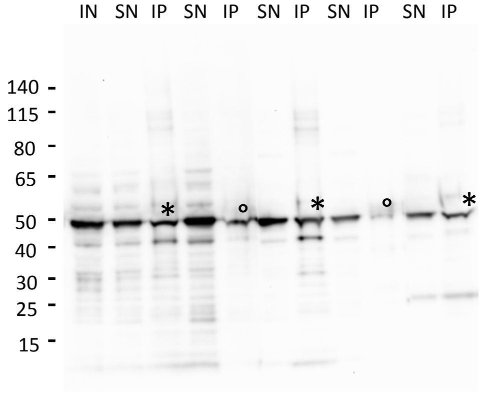

Transfection	GFP-VAMP7 (human)				
IP antibody	158.2	IgGM	TG50	IgGR	GFP
IP beads specificity	anti-mouse		anti-rabbit		
WB (primary Ab)	TG50 (AP)		158.2		GFP
WB (IgG IRDye 800 secondary Ab)	anti-rabbit		anti-mouse		

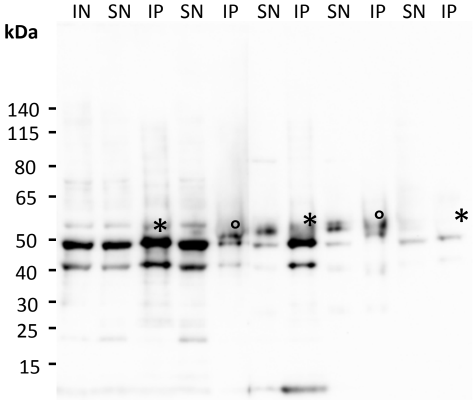

* = GFP-VAMP7 (expected size: ~50 kDa)

° = Absence of band at GFP-VAMP7 size

D

Cortex lysate	WT						VAMP7 KO				
IP antibody		TG50		IgGR				TG50		IgGR	
IP beads	anti-rabbit IgG										
WB (primary Ab)	158.2										
WB (secondary Ab)	anti-mouse IgG IRDye 800										
	IN	SN	IP	SN	IP		IN	SN	IP	SN	IP

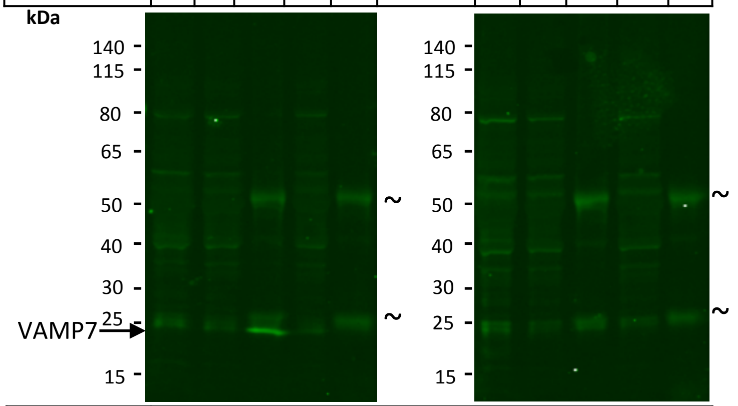

WB (primary Ab)	TG50
WB (secondary Ab)	anti-rabbit IgG HRP

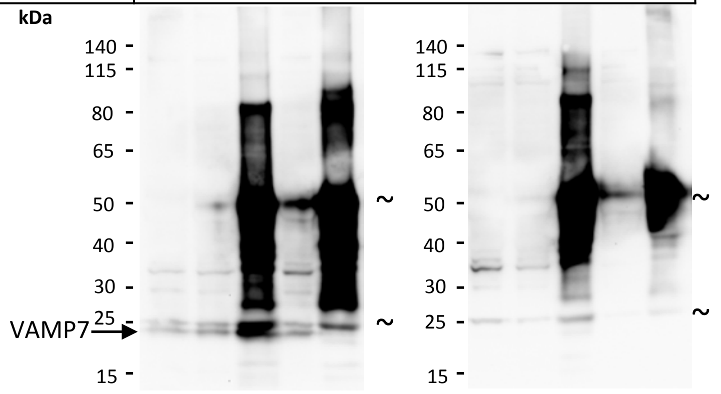

~: immunoglobulins
