# Supplementary material for: Comparative study of commercially available and homemade anti-VAMP7 antibodies using CRISPR/Cas9-depleted HeLa cells and VAMP7 knockout mice
Source: F1000Res. 2019 Feb 7;7:1649. Originally published 2018 Oct 16. [Version 2] doi: 10.12688/f1000research.15707.2 (PMC6376254; doi:10.12688/f1000research.15707.2)
Supplement: Peer review history

# Peer review: Referee response for version 2

**DOI:** 10.5256/f1000research.19822.r44161

**Published:** 2019-02-13

**Authors:** Alison H. Banham

**Affiliations:** 1 Nuffield Division of Clinical Laboratory Sciences, Radcliffe Department of Medicine, John Radcliffe Hospital, University of Oxford, Oxford, UK

The amended version addresses the points raised in my previous review. It was nice to see that the TG50 antibody was able to detect the endogenous VAMP7 protein by Western Blotting, suggesting that it is indeed more sensitive than 158.2.

I have read this submission. I believe that I have an appropriate level of expertise to confirm that it is of an acceptable scientific standard.

# Peer review: Referee response for version 1

**DOI:** 10.5256/f1000research.17141.r40504

**Published:** 2018-11-27

**Authors:** Marc G. Coppolino, Olivia Grafinger

**Affiliations:** 1 Department of Molecular and Cellular Biology, University of Guelph, Guelph, ON, Canada

This study assessed the performance of four monoclonal and four polyclonal antibodies to VAMP7. Six commercially available antibodies, and two antibodies generated by the researchers, were compared by Western blot, immunocytochemistry and immunoprecipitation. Lysates from HeLa and HeLa-VAMP7 knockout cells, and extracts from wildtype and VAMP7 knockout mouse brain tissue were used to examine specificity and background of the antibodies. The findings indicate some of the antibodies outperform comparators in blotting, immunocytochemistry, with reduced background and higher specificity, and perform well in immunoprecipitations. This work offers careful characterization of VAMP7 antibodies for use by other researchers in making decisions about antibody use for analytical studies of this protein.

 The study is well conceived and executed. The approaches used are suitable, and the description of work is adequately detailed. Data are clearly presented, and for the most part conclusions are reasonable.

 There are a few points that should be addressed to strengthen the study:

1. For the Western blot analyses, the antibody concentrations used (ug/ml) should be provided to allow more accurate comparison of antibody performance.
2. In the IF specificity index graph (Fig. 2C), it appears that the authors only measured the intensity profile in one cell for each antibody. These results would be more convincing if the intensity profiles of multiple cells were shown, as there can be variations in staining patterns that can arise from different cell morphologies, etc.
3. It is not clear why some of the antibodies, which performed reasonably well in Western blot and immunocytochemistry (Creative Diagnostics, Synaptic Systems), were not analysed in immunoprecipitations. An explanation for this, or information on their performance in this regard, would be useful.

We have read this submission. We believe that we have an appropriate level of expertise to confirm that it is of an acceptable scientific standard.

# Peer review: Referee response for version 1

**DOI:** 10.5256/f1000research.17141.r39566

**Published:** 2018-10-26

**Authors:** Alison H. Banham

**Affiliations:** 1 Nuffield Division of Clinical Laboratory Sciences, Radcliffe Department of Medicine, John Radcliffe Hospital, University of Oxford, Oxford, UK

This study has compared the specificity of a panel of antibodies against VAMP7, a member of the SNARE protein family. Using CRISPR/Cas9 to create a HeLa-VAMP7 knockout cell line together with knockout mouse tissues the authors effectively demonstrate that the panel of antibodies are able to recognise the endogenous VAMP7 protein, the majority performing effectively in both WB and IF. Image analysis was performed to profile the signal-to-noise index in both techniques. While this does provide quantitative data with which to prioritise the antibodies, I reached the same conclusions without needing the profiling data. This approach may indeed have utility in other projects but perhaps consideration should be given to incorporating a measure for antibody sensitivity. Antibodies with similar specificity and different levels of sensitivity may have a similar specificity index score but may not be equally useful. Two antibodies were also validated for their ability to IP recombinant orthologous VAMP7 proteins and one for IP of the endogenous protein. This is a useful comparative study that will be of interest to other researchers sourcing antibodies to further characterise this molecule.

**Comments:**

 1) P3: For clarity I would suggest rephrasing ‘Invalidation’ of VAMP7 as inactivation, knockout or deletion.

 2) P5 and P9 bottom left column: Readers are referred to Table 3 for dilutions of primary antibodies yet this information seems to be provided in Table 1.

 3) While the image analysis provides quantitative information, the best antibodies identified using this approach were also easily identifiable by looking at the WB and IF images presented. With regard to the WB data in Figure 1, if just using the WB specificity index then there potentially appeared to be relatively little to choose between the two best antibodies. However, by eye, TG50 appears to have much greater sensitivity for VAMP7 detection, yet this distinction is not conveyed by a score which focuses on signal-to-noise.

 4) In Figure 2 – there is clearly significant heterogeneity in the distribution of VAMP7 both within and between individual HeLa cells. It is not clear from the methods whether data from individual cells or a large number were used to generate the IF specificity index and this information should be provided. The data would be more robust if multiple cells were sampled.

 5) Figure 2 indicates Cell Signaling 14811 performed poorly, however on the supplier’s site this antibody (D4D5J) is not recommended for IF and it is clearly stated by the authors in the results that an alternative (D8Y1R) was used. This might just be an error in labelling on the figure? However, the text describing these IF data also refers to the antibody being used as 14811 (which is D4D5J). This needs to be clarified and it would be helpful within the manuscript and figures to consistently refer to either the clone names or the catalogue number when describing an antibody.

 6) In the discussion the authors correctly comment that not using individually optimised conditions for each antibody in IF may disadvantage some of the reagents. I think it would have been more helpful to other researchers wanting to know which antibody works best for IF to be able to compare images obtained using an optimal staining protocol, such as that determined by each manufacturer. 

 7) In the discussion the authors comment on the sensitivity of 158.2 being insufficient to detect low levels of the endogenous VAMP7 protein by WB. When TG50 seemed to be more sensitive in WB analysis then it would perhaps have been the more obvious choice for the WB detection antibody. This point could be added to the discussion.

I have read this submission. I believe that I have an appropriate level of expertise to confirm that it is of an acceptable scientific standard, however I have significant reservations, as outlined above.
